# Characterization and Kinetic Studies of Poly(vinylidene fluoride-co-hexafluoropropylene) Polymer Inclusion Membrane for the Malachite Green Extraction

**DOI:** 10.3390/membranes11090676

**Published:** 2021-08-31

**Authors:** Jillin Ai Lam Soo, Muaz Mohd Zaini Makhtar, Noor Fazliani Shoparwe, Tunmise Ayode Otitoju, Mardawani Mohamad, Lian See Tan, Sanxi Li

**Affiliations:** 1 Faculty of Bioengineering and Technology, Jeli Campus, Universiti Malaysia Kelantan, Jeli Kelantan 17600, Malaysia; jillin_soo@yahoo.com (J.A.L.S.); timopd1@gmail.com (T.A.O.); mardawani.m@umk.edu.my (M.M.); 2 Malaysia–Japan International Institute of Technology, Universiti Teknologi Malaysia, Kuala Lumpur 54100, Malaysia; tan.liansee@utm.my; 3 Bioprocess Technology Division, School of Industrial Technology, Universiti Sains Malaysia, Pulau Pinang 11800, Malaysia; 4 School of Materials Science and Engineering, Shenyang University of Technology, Shenyang 110870, China; 5 School of Environmental and Chemical Engineering, Shenyang University of Technology, Shenyang 110870, China; lisx@sut.edu.cn

**Keywords:** polymer inclusion membrane, extraction, malachite green, poly(vinylidene fluoride-co-hexafluoropropylene), bis-(2-ethylhexyl) phosphate, kinetic study

## Abstract

Textile industry effluent contains a high amount of toxic colorants. These dyes are carcinogenic and threats to the environment and living beings. In this study, poly(vinylidene fluoride-co-hexafluoropropylene) (PVDF-co-HFP) was used as the based polymer for PIMs with bis-(2-ethylhexyl) phosphate (B_2_EHP) and dioctyl phthalate (DOP) as the carrier and plasticizer. The fabricated PIMs were employed to extract the cation dye (Malachite Green; MG) from the feeding phase. PIMs were also characterized by scanning electron microscopy (SEM), atomic force microscope (AFM), contact angle, water uptake, Fourier-transform infrared spectroscopy (FTIR) and ions exchange capacity. The performance of the PIMs was investigated under various conditions such as percentage of carrier and initial dye concentration. With permeability and flux values of 0.1188 cm/min and 1.1913 mg cm/min, PIM produced with 18% *w/w* PVDF-co-HFP, 21% *w/w* B_2_EHP, 1% *w/w* DOP and 40% *w/w* THF and was able to achieve more than 97% of MG extraction. The experimental data were then fitted with a pseudo-second-order (PSO) model, and the calculated R^2^ value was ~0.99. This shows that the data has a good fit with the PSO model. PIM is a potential alternative technology in textile industry effluent treatment; however, the right formulation is crucial for developing a highly efficient membrane.

## 1. Introduction

Textile effluents contain vat dyes, nitrates, acetic acid, soaps, chromium compounds and heavy metals such as arsenic, lead, copper, cadmium, mercury, nickel and cobalt which render the effluent highly toxic and carcinogenic to living things [1]. Exposure to these textile effluents could result in the dysfunction of organs, specifically the kidney, reproductive system, liver, brain and central nervous system [2,3]. For instance, malachite green (MG) is extensively used in the textile industry [4,5]. The discharge of MG effluent gives undesirable color to the effluent, and it reduces the penetration of sunlight into the river or lake which threatens the life of the aquatic ecosystem with hypoxiation. Likewise, the nitrogen compound in MG is carcinogenic, genotoxic, mutagenic and teratogenic to living organisms [6]. The conventional treatments such as adsorption, ion exchange, aerobic and anaerobic, oxidation, coagulation and flocculation are ineffective to treat textile wastewater. This is due to the chemical and physical properties of the dye where the presence of several benzene rings has high resistance towards microbial attachment, fixation and fastness (stability in light and washing) [7]. Therefore, researchers are keen for an alternative treatment and this led to the introduction of the polymer inclusion membrane (PIM). PIM is a flat sheet liquid membrane (LM) that is fabricated from a mixture of the base polymer, extractant/carrier, plasticizer and solvent solutions. PIM separates two different fluid phases (feeding and receiving) and at the same time links the two fluid phases for selective permeation [8]. PIM is an energy-saving technology with low operating costs and a simple operation mode [9], top up with a higher diffusion coefficient and selectivity and flexibility properties. In addition, compared to other LM, PIM has better stability and a longer lifetime [10]. PIM has proven to be effective (with more than 90% of removal) in the extraction of a wide range of components such as metals (e.g., chromium, zinc, copper, silver, nickel, cobalt, mercury, gold), dyes (e.g., malachite green, methylene blue, reactive orange 16) and other charged ions (e.g., phenol, picloram).

Foremost, the formulation of the membrane is the key component to fabricate the membrane with high performance and mechanical strength. Extensive research had been studied on the formulation of PIM with base polymer, cellulose triacetate (CTA) and poly(vinyl chloride) (PVC) (Table 1). However, studies showed that CTA- and PVC-based PIMs have low performance in long extraction periods [11,12]. Thus, researchers have been looking into an alternative base polymer such as poly(vinylidene fluoride-co-hexafluoropropylene) (PVDF-co-HFP). This is because PVDF-co-HFP has higher stability than other base polymers. Furthermore, it has better extraction and transport efficiency compared to CTA and PVC [11]. PVDF-co-HFP is commonly used in lithium batteries and fuel cells due to high ionic conduction and mechanical support [13]. This shows that PVDF-co-HFP is a potential base polymer for PIM technology. 

To our best knowledge, the study of PVDF-co-HFP in PIM is limited to metal extraction. So far, there has been no study on dye removal using PVDF-co-HFP. Therefore, this research aims to study the formulation of PVDF-co-HFP-based PIM with a B_2_EHP carrier, DOP plasticizer and THF solvent. Moreover, the performance of the PIM will be determined with MG extraction under various conditions such as different percentages of the carrier and initial dye concentration followed by characterization studies using Fourier transform infrared spectroscopy (FT-IR), scanning electron microscopy (SEM), atomic force microscopy (AFM), contact angle, water uptake and ionic exchange capacity (IEC). The kinetic parameters of the MG extraction were also evaluated in this research for an in-depth understanding of the mechanism of the MG extraction.

## 2. Materials and Methods

### 2.1. Materials

**Figure 1:**
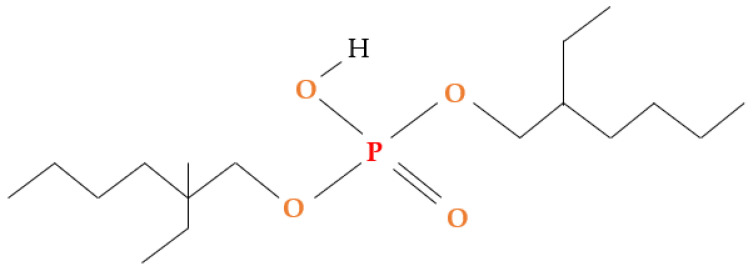
Chemical structure of bis-(2-ethylhexyl) phosphate (B_2_EHP).

**Figure 2:**
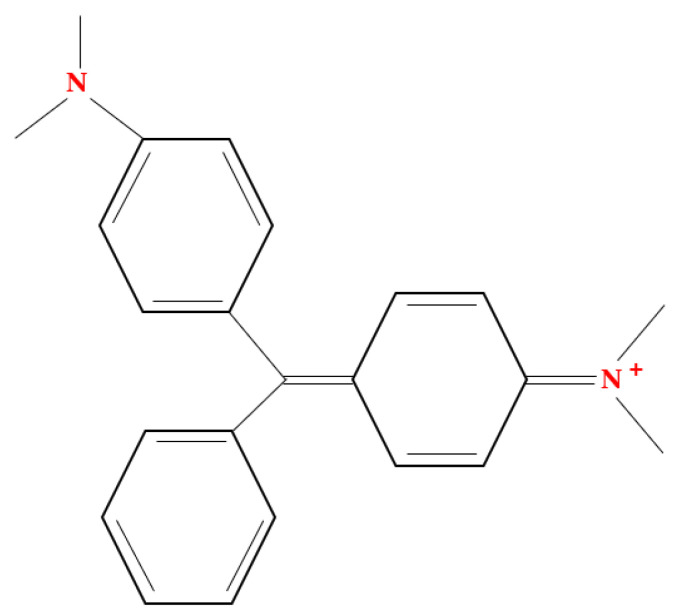
Chemical structure of malachite green (MG).

Poly(vinylidene fluoride-co-hexafluoropropylene) (PVDF-co-HFP) (C_5_H_2_F_8_), bis-(2-ethylhexyl) phosphate (B_2_EHP) (C_16_H_35_O_4_P) (Figure 1), dioctyl phthalate (DOP) (C_24_H_38_O_4_), tetrahydrofuran (THF) (C_4_H_8_O), 65% nitric acid and malachite green (MG) (C_23_H_25_CIN_2_) (Figure 2) were supplied by Sigma-Aldrich (St Louis, MO, USA). A total of 38% hydrochloric acid, phenolphthalein were supplied by HmbG Chemicals (Hamburg, Germany). Sodium hydroxide was supplied by R&M Chemicals (London, UK) and deionized water.

### 2.2. Fabrication of PIMs

A predetermined amount of PVDF-co-HFP powder was dissolved in THF, followed by the addition of solutions containing B_2_EHP and DOP. The solution was stirred for 4 h at 400 rpm using a stirring hotplate at room temperature until a clear and homogenous solution was obtained. The membrane was cast with a membrane casting machine (thickness of 0.15 mm) and left to dry overnight in a fume hood at room temperature. The dried membrane was then peeled off and cut into a desired circular shape (~3.0 cm in diameter). The membrane was rinsed several times with distilled water to remove the excess solvent before further analysis was conducted. The procedure was repeated with different formulations of PIMs (Table 2). M1 acts as the control as it does not contain the carrier whereas M2, M3, M4, M5 and M6 signify PIMs with varying percentages of the carrier.

### 2.3. H-Cell Device Set Up

**Figure 3:**
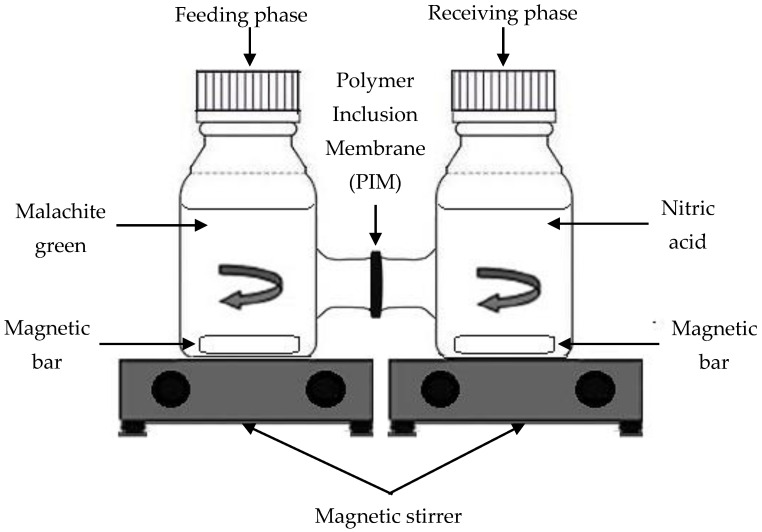
H-cell device set up.

MG extraction was performed using an H-cell device (Figure 3). In brief, the MG ions were extracted from the feeding phase into the receiving phase passing through the membrane. During the extraction process, continuous agitation was provided to ensure both solutions were in homogenous conditions. The diameter and distance of the linkage hollow tube are ~3.0 cm and ~8.0 cm, respectively. The device was separated into two compartments (feeding and receiving phase). The MG solution was filled in the feeding phase, and 1M of 67% nitric acid was filled in the receiving phase.

### 2.4. Performance Studies for the MG Extraction

**Figure 4:**
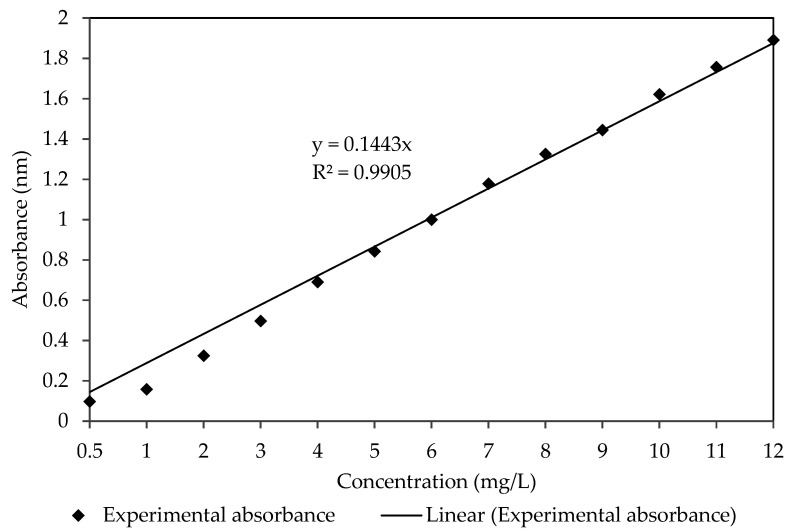
MG calibration curve.

The feeding phase was filled with 120 mL of 10 mg/L MG solution. During the MG extraction process, both the feeding and receiving solutions were continuously stirred at 350 rpm using a magnetic stirrer for 4 h. The experiment was conducted at room temperature at 1 atm. A total of 1 mL of the sample was collected from the feeding phase using a micropipette after every 30 min. Then, the absorbance of MG was determined by the UV-Vis spectrophotometer at 617 nm wavelength, and the concentration of MG was calculated based on the calibration curve (Figure 4) [35]. This experiment was repeated by varying the PIM compositions. Based on the results obtained, the membrane with the best performance was further tested with different initial concentrations of MG solutions (2, 4, 6, 8, 10 and 12 mg/L). The percentage of extraction efficiency (*E*%) was calculated using Equation (1):

(1) $$E\%=\frac{{\left(dye\right)}_{i}-{\left(dye\right)}_{f}}{{\left(dye\right)}_{i}}\times 100\%$$

where ${\left(dye\right)}_{i}$ is the initial dye concentration in the aqueous phase (mg/L); ${\left(dye\right)}_{f}$ is the final dye concentration after the extraction in the aqueous phase (mg/L).

### 2.5. Transport Kinetics

The permeability and flux were calculated from the rate constant value from Section 2.7. The permeability coefficient (*P*) can be calculated from Equation (2):

(2) $$P=\frac{V}{A}k$$

where V was the volume of feeding solution (cm^3^); A was the effective area of PIM (cm^2^).

Initial flux (J) can be computed by the Equation (3):

(3) $$J=P\times C_{0}$$

where $C_{0}$ is the initial concentration of MG in the feeding solution (mg/L).

### 2.6. Characterization of PIMs

#### 2.6.1. Scanning Electron Microscopy (SEM)

The surface morphology of the membranes was observed by scanning electron microscopy (SEM) using the HITACHI Tabletop Microscope instrument (TM-3000-Japan) by HITACHI (Tokyo, Japan). The membrane was cut into 5 mm × 5 mm in size and coated with gold. The surface morphology of the membranes was then analyzed under 1000× magnification with 10 kV acceleration voltage [36].

#### 2.6.2. Atomic Force Microscopy (AFM)

Membrane uniformity and roughness of the fabricated PIMs surface were determined using AFM (Model XE-100) by Park Scientific Instrument (Suwon, Korea) [37]. The membrane was visualized in two-dimensional and three-dimensional form with three sizes (1.0 × 1.0 μm, 5.0 × 5.0 μm and 10.0 × 10.0 μm) [38].

#### 2.6.3. Contact Angle (CA)

The hydrophobicity of the membrane was determined using a contact angle goniometer (Model: OCA15plus) by DataPhysics Instruments GmbH (Filderstadt, Germany). The contact angle was measured by dropping 1 μL of distilled water on the surface of the membrane through a needle tip attached to the goniometer. The magnified image of the water droplet was then observed using a digital camera, and the CA readings were obtained at 10 s after the deposition of the water droplet on the surface of the dried membrane. The contact angles of each membrane at 5 different spots were recorded, and their mean values were calculated [39].

#### 2.6.4. Water Uptake

Water uptake was performed to determine the wettability of the membranes. The membrane was cut into 2 cm × 2 cm in size. Then, the weight of each membrane was determined using an electronic balance (ME204E) by Mettler Toledo (Greifensee, Switzerland) and the membrane was immersed in distilled water for 30 min. Subsequently, the membrane was removed from the distilled water, and the excess liquid on the membrane was gently dapped by a tissue towel. The membrane was then weighed for the second time to determine the weight change after the absorption of distilled water. The water uptake of the membrane was calculated using Equation (4) [40].

(4) $$Water\;uptake=\frac{W_{wet}-W_{dry}}{W_{dry}}$$

where $W_{wet}$ is the weight of the wet membrane after the absorption of distilled water, and $W_{dry}$ is the weight of the dry membrane before immersion in distilled water.

#### 2.6.5. Fourier Transform Infrared Spectroscopy (FTIR)

FTIR was used to determine the functional groups of the fabricated samples and PIMs by comparing the analyzed results and the existing functional groups standard such as alkane (-CH and -CH_2_ bonds), alky halide (-C-F, -CF_2_ and -CF_3_ bonds), aromatic group, ester, alcohol (-P-OH bond), carbonyl group (C=O bond), hydroxyl group (O-H bond) and carbon-carbon bond (C-C and C-C-C bond) [41,42,43]. FTIR was performed using the iZ10 FTIR Spectrometer by Thermo Fisher Scientific (Massachusetts, United States). The spectrum recorded was between 400–4000 cm^−1^ wavenumber using 16 scans at a resolution of 4 cm^−1^. The samples were tested by transmission method, and the spectra were analyzed using the OMNIC software [44].

#### 2.6.6. Ion Exchange Capacity (IEC)

The IEC of the membranes was measured using the titration method. The membrane was cut into 2 cm × 2 cm and soaked in 1 mol/dm^3^ HCl for 24 h. Then, the membrane was removed and rinsed with distilled water to remove the excess HCl on the surface of the membrane. The membrane was subsequently immersed in the 1.0 mol/dm^3^ NaCl solution for another 24 h. After 24 h, the membrane was removed, and the remaining solution was titrated with 0.01 mol/dm^3^ NaOH solution with few drops of phenolphthalein as an indicator [45]. The IEC of the membrane was calculated by using Equation (5):

(5) $$IEC=\frac{ab}{W_{dry}}$$

where a is the concentration of titrated NaOH solution (mol/dm^3^); b is the volume of NaOH solution (dm^3^) and $W_{dry}$ is the dry weight of the membrane (g).

### 2.7. Kinetic Studies

The process of MG extraction is simple, but it involves a complex mechanism. To describe the nature and mechanism applied in this technology, a kinetic modeling study was necessitated. Kinetic models can describe the interaction between the absorbate and the absorbent such as chemisorption or physisorption. The results obtained from the different initial MG concentrations were fitted using nonlinear pseudo-first-order (in Equation (6)) and pseudo-second-order (in Equation (7)) [46]. The parameter estimation of the kinetic model was obtained from nonlinear least-squares regression using the Levenberg–Marquardt method Polymath R Version 6.2 by CACHE Corporation (Austin, TX, USA) software. The software estimated the value of parameters within the non-linear equations from the experimental results by minimizing the sum of square error [47]. The validation of the model was analyzed using variance as shown in Equation (8).

(6) $$C=C_{e}-\exp\left(-k_{1}t\right)\left(C_{e}-C_{0}\right)$$

(7) $$C=C_{e}+\frac{1\;}{k_{2}t-\frac{1}{\left(C_{e}-C_{0}\right)}}$$

(8) $$\sigma^{2}=\frac{\sum {\left(x-\bar{x}\right)}^{2}}{n-1}$$

where $C_{e}$, $C_{0}$, k, ∑, *x*, $\bar{x}$ and n refer to the concentration at equilibrium, initial concentration, rate constant (mg/g), summation of data, experimental data, mean of the experimental data and sample size, respectively. The coefficient of determination “*R^2^*” value was used to determine the best-fitted model for the extraction process where the maximum value of “*R^2^*” is considered more favorable [48]. The aim is to validate and compare the results obtained from this experiment to the result calculated using the kinetics models.

## 3. Results and Discussion

### 3.1. Parameters of the Extraction of MG

#### 3.1.1. Effect of Carrier Percentage

The effect of carrier composition (B_2_EHP) was studied by altering the composition of the B_2_EHP (6, 9, 15, 18 and 21% *w/w*) for the extraction of 10 mg/L of MG as shown in Figure 3. B_2_EHP is an acidic extractant that is highly efficient in transporting cations across the hydrophobic membrane [36]. B_2_EHP is also widely known as di-2-ethylhexyl hydrogen phosphate (D_2_EHP) or di-2-ethylhexyl hydrogen phosphate acid (D_2_EHPA). B_2_EHP was chosen due to its low solubility in aqueous solutions and chemical stability. The extraction chemistry was described using Equation (9). Several components were involved in the reaction such as MG, B_2_EHP and neutral ion-pair complexes.

(9) $$MG^{+}{}_{\left(aq\right)}+{\left[RH_{2}\right]}_{\left(org\right)}\to {\left[MG\left(RHR\right)\right]}_{\left(org\right)}+H^{+}{}_{\left(aq\right)}$$

where $RH_{2}$ is the carrier (B_2_EHP); $MG^{+}$ is the MG ion and $\left[MG\left(RHR\right)\right]$ is the neutral ion-pair complex.

**Figure 5:**
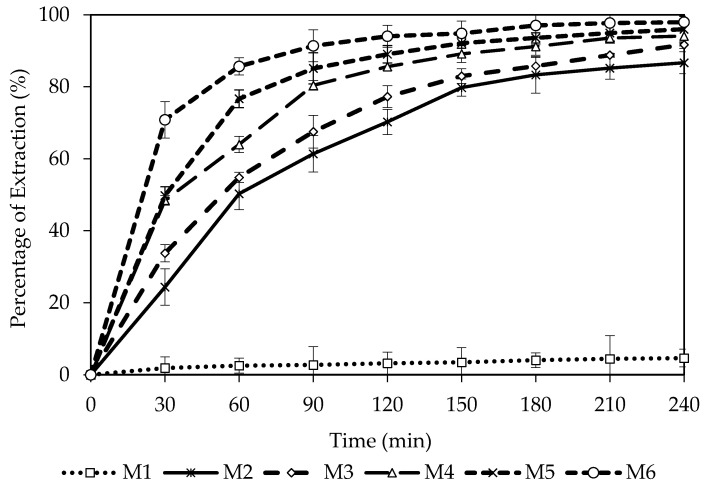
Percentage of extraction of M1, M2, M3, M4, M5 and M6.

As observed in Figure 5, M1 shows significantly low extraction of MG as compared to other membranes (with carrier). A similar result was also reported by Pérez-Silva et al. [49] who studied the removal of phenol with PIM. They confirmed that a membrane without a carrier will only be able to transport a low percentage of phenol. On the contrary, the performance of the membrane surged up to 86.68% of MG extraction with M2 and was followed by a steady increment as the amount of carrier increased. The best performance was achieved with M6 with 97.97% of MG extraction compared to M3 (91.71%), M4 (94.09%) and M5 (96.00%). The extraction efficiency also reflects on the permeability, *P*, and flux, *J* of the membrane. Table 3 shows the effect of the percentage of the carrier against the permeability and flux for the membranes. Comparable trends are observed for the *P* and *J* values and the performance of MG extraction. M1 presents a low *P* and *J* values of 0.0068 cm/min and 0.0643 mg cm/min, respectively. Likewise, a gradual increment of both values is noticed for M2 to M6 (0.0255 cm/min to 0.1188 cm/min; 0.2540 mg cm/min to 1.1913 mg cm/min). This signifies that the carrier plays a significant role in facilitating the transportation of ions across the membrane [36]. When more carriers are incorporated into the membrane, there is formation of more capacity for the MG^2+^ -B_2_EHP complex; thus, the rate of MG extraction was enhanced. This result is in agreement with other researchers on the PIM extraction/removal of methylene blue, indium (In(III)), chromium (Cr(III)) and copper (Cu(II)) with the D_2_EHPA carrier [8,50,51,52]. Hence, M6 with 21% B_2_EHP content was selected as the PIM with the best performance for further studies.

#### 3.1.2. Effect of Initial Dye Concentration

**Figure 6:**
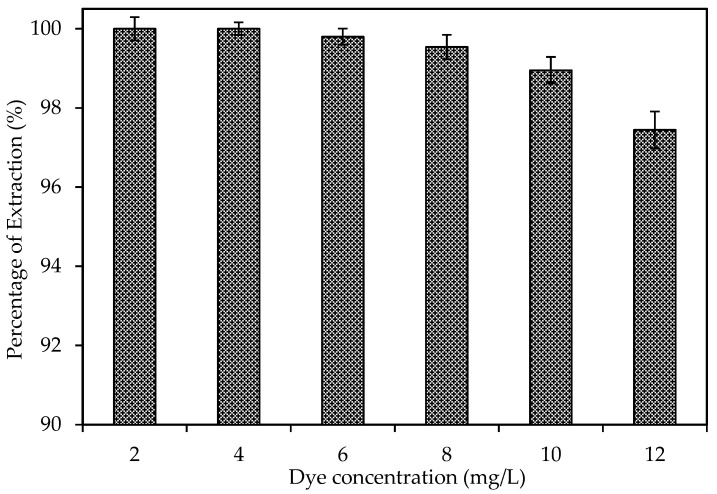
Dye extraction of M6 membrane at different dye concentrations.

The effect of initial dye concentration varied (2 mg/L to 12 mg/L), while other variables were maintained constant. Figure 6 presents the influence of the initial concentration on dye extraction. Observably, increasing the dye concentration from 2 mg/L to 12 mg/L decreases the rate of removal. As observed, the best dye extraction (>99.99%) was achieved using 2 mg/L and 4 mg/L of dye concentration. Meanwhile, 99.80%, 99.54%, 98.95% and 97.45% were achieved using 6 mg/L, 8 mg/L, 10 mg/L and 12 mg/L, respectively. Table 4 also shows the influence of the initial concentration on permeability and flux. As observed, there was a slight decrease in permeability with the increment in dye concentration. The reduction in the extraction efficiency can be related to the saturation of the carrier on the surface of the membrane which resulted in a reduction in the diffusion boundary layer and hence slower transport kinetics [53]. This finding is consistent with the outcome of other research [36,51,54,55].

### 3.2. FTIR Analysis

**Figure 7:**
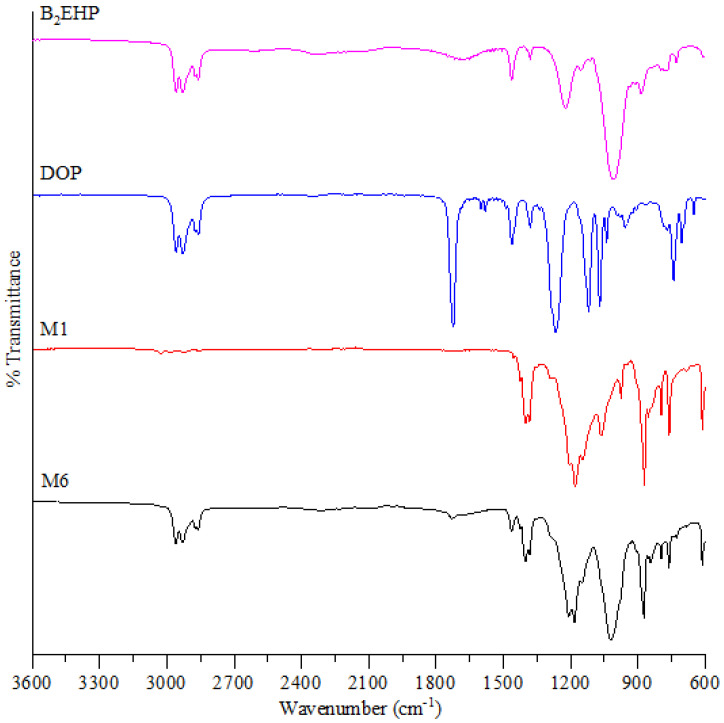
FTIR spectrum analysis on B_2_EHP, DOP, M1 and M6.

**Figure 8:**
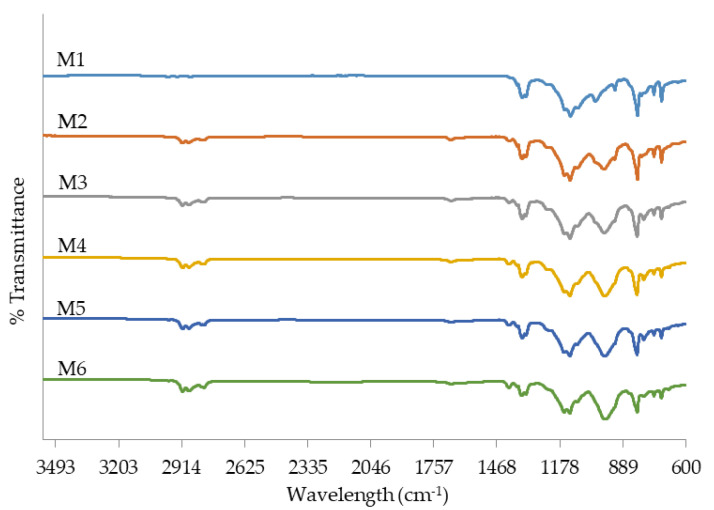
FTIR spectrum analysis on M1, M2, M3, M4, M5 and M6.

FTIR was employed to determine the functional groups of the blank membrane and PIMs. The FTIR spectra are shown in Figure 7 which gives the comparison of B_2_EHP, DOP, M1 and M6. B_2_EHP shows a characteristic intense peak at 1019.71 cm^−1^ which was contributed by the P-O-C bond. The peaks at 1424.20–1382.67 cm^−1^ and 727.45 cm^−1^ correspond to sp^3^ C-H bending and C-H_2_ rocking, respectively. Weak peaks obtained at 2958.92–2860.40 cm^−1^ correspond to the sp^3^ C-H bond and were found in both B_2_EHP and DOP. DOP also gave rise to weak and sharp peaks at 1728.32 cm^−1^ and 1462.78 cm^−1^ that relate to the presence of the C=O bond and C=C stretching in the ring. Characteristic peaks exhibited by the blank PVDF-co-HFP membrane (M1) are at 1208.87–1149.83 cm^−1^, 795.59 cm^−1^ and 612.8 cm^−1^ which correspond to C-F stretching, C-F_3_ stretching vibration and C-F_2_ bending, whereas peaks at 872.58 cm^−1^ and 761.71 cm^−1^ are related to the C-C stretching and C-H_2_ rocking vibration. As in Figure 7, it can be observed that the characteristic of bands of B_2_EHP, DOP and PVDF-co-HFP exist in PIMs (M2-M6) with no displacement. The spectra of PIMs suggested that carriers and plasticizers were entangled in the polymeric matrix without any chemical changes; hence, the carrier is free to interact with the ions in the solution [56]. Likewise, a broad peak at 1684.05 cm^−1^ that arises from O-H stretching in B_2_EHP disappeared in PIM. This gives the idea that the O-H group was involved in the formation of the membrane via the Van der Waals force of attraction or hydrogen bond [27]. The analyzed peaks and functional groups are detailed out in Table 5, and FTIR spectra for M1, M2, M3, M4, M5 and M6 are displayed in Figure 8.

**Figure 9:**
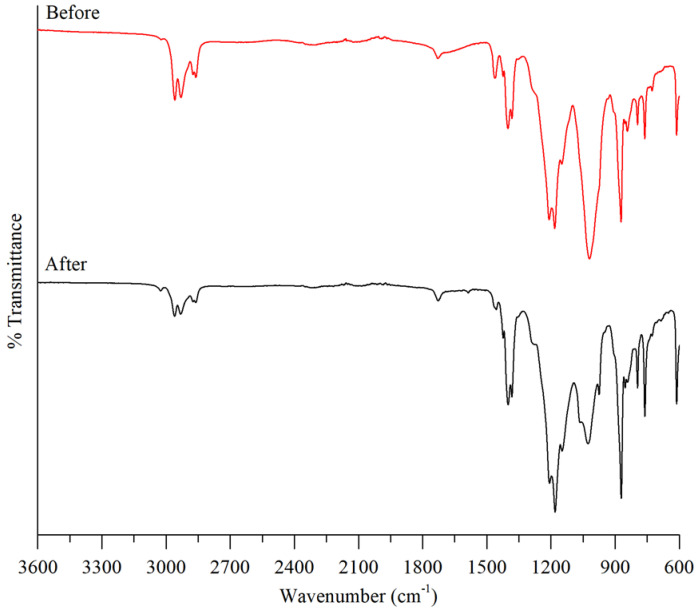
FTIR spectrum before and after extraction of MG.

The FTIR spectra for M6 before and after the MG extraction were compared as shown in Figure 9. There is a significant reduction in the intensity of the peaks at 2958.92–2860.40 cm^−1^ (sp^3^ C-H bond) and 1019.71 cm^−1^ (P-O-C bond) after the extraction. This proposes that these peaks are related to the active sites of the carriers which are responsible for the extraction of MG ions. The reduction in the intensity of the peaks can be explained by the decrease in the amount of vacant active sites of the carrier after the extraction process of the weak peaks which are detected after extraction membrane. On the contrary, there is also an increment in the intensity of spectra at 1208.87–1149.38 cm^−1^, 872.58 cm^−1^ and 795.59–612.8 cm^−1^ which originated from the presence of the C-N stretching aromatic amine, C-N stretching amine and para disubstituted and monosubstituted benzene ring. The increment of the C-N bonds proved that MG ions adhered on the surface of the membrane during the extraction process, and it is found that O-H bonds were formed between the carrier and MG ion which led to the formation of the peak at 1424.20–1382.67 cm^−1^.

### 3.3. Surface Morphology of PIMs (SEM Analysis)

**Figure 10:**
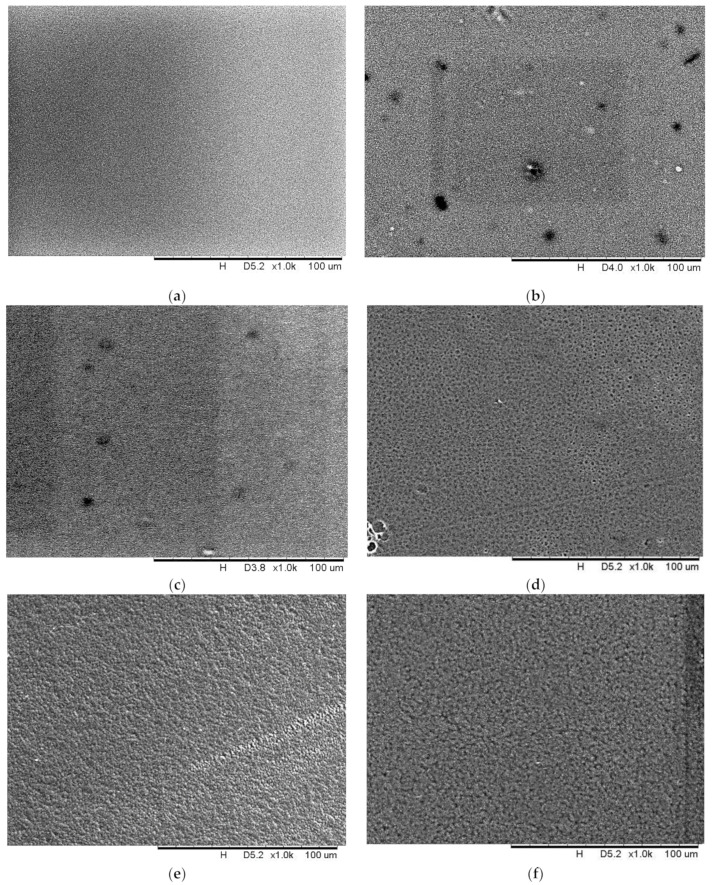
SEM image at 1000× of (**a**) M1, (**b**) M2, (**c**) M3, (**d**) M4, (**e**) M5 and (**f**) M6.

To examine the influence of the carrier percentage in PIMs, SEM was applied to determine the surface morphology of the membranes. Figure 10 shows different surface morphologies of M1, M2, M3, M4, M5 and M6. It can be observed that uneven dark spots were homogeneously distributed on the surface of all the membranes. From Figure 10a, tiny dark spots were observed on the smooth surface of the blank PVDF-co-HFP membrane (M1) which is due to the formation of pores after the evaporation of the solvent. Mahendrakar and Anna [57] reported similar phenomena which were attributed to the semi-crystalline nature of the membrane. However, as the percentage of carriers increases, the dark spots become distinct and visible. This is due to the inclusion of the carrier which increased the porosity of the membrane as well as the number of pores found on the surface of the membrane. These carrier molecules are useful in facilitating the transportation of the cations by forming continuous liquid domains across the PIM [58]. According to Gherasim et al. [58], these liquid domains can also be the “liquid pores” that enhance the transportation process. In fact, with the addition of a plasticizer, the ionic conductivity of the membrane was further improved with the formation of interconnecting linkages between the liquid pores [59]. This enables the efficient transport of ions across the membranes. Likewise, the ionic conductivity was also improved by reducing the crystallinity of the membranes and increasing the amorphous nature of the membrane.

### 3.4. AFM Analysis

**Figure 11:**
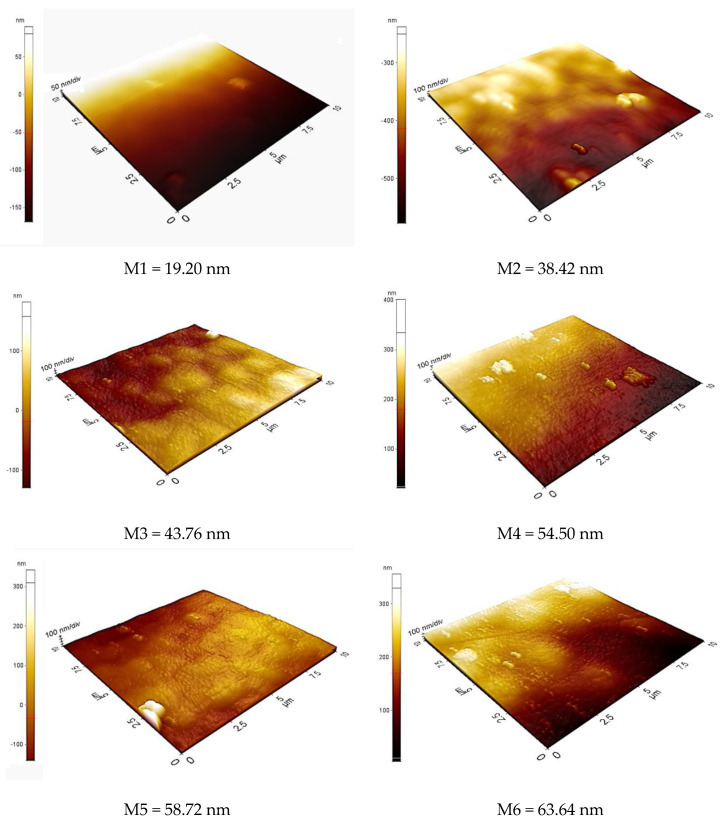
AFM image of M1, M2, M3, M4, M5 and M6.

AFM was applied to characterize the surface morphology of the membrane by determining the surface roughness of the membrane which is crucial for understanding the transportation of ions across the polymeric membrane. The 3D AFM images in Figure 11 indicate the surface of the M1, M2, M3, M4, M5 and M6. From Figure 11, it can be observed that the surface roughness (Ra) of M1 to M6 increases gradually from 19.20 nm to 63.64 nm. Compared with other membranes, M1 has the smoothest surface with the lowest surface roughness. Homogenous distributions of dark regions were found on the surface of the blank membrane due to the formation of pores which is a result of solvent evaporation. These pores act as the vessels for the encasement of carriers and plasticizers. Increasing roughness was noticed for M2, M3, M4, M5 and M6 with the roughness values of 38.42 nm, 43.76 nm, 54.50 nm, 58.72 nm and 63.64 nm, respectively. This signifies that the carrier and plasticizer have a close relationship with the improvement of surface roughness. As more carriers are added to prepare the membrane, the surface becomes rougher. The embedded carriers are known as liquid drops (carrier) which penetrate the membrane surface and are thus responsible for the cation sorption in PIMs [60]. These carriers crystallized at the surface of the membrane, forming pores and thus forming a rough surface [61]. In addition, rougher surfaces also provide a larger total surface area for the extraction to take place. Therefore, the transport of ions across the membrane and extraction efficiency were enhanced as the percentage of the carrier increased.

### 3.5. Contact Angle, Thickness and Water Uptake

The surface properties of PIMs can greatly affect the permeability and flux of the membranes. Contact angle, thickness and water uptake were employed in these studies. The membrane with a higher contact angle (>90°) is determined as hydrophobic, whereas the membrane with a lower contact angle (<90°) is hydrophilic [62,63]. The measured values of contact angle, water uptake and thickness for the membranes were tabulated in Table 6. From Table 6, M1 is the most hydrophobic membrane among others with the contact angle of 153.48°, whereas M6 is the least hydrophobic membrane with a contact angle of 95.54°. A declining trend was observed for the membranes as the percentage of carriers increased. As observed, the M1 membrane exhibited the highest contact angle. This can be explained by the introduction of the HFP functional group which enhances the fluorine content in the base polymer and subsequently increases the hydrophobic properties of the PVDF-co-HFP [64,65]. However, when the hydrophilic carriers were filled into the pores of the hydrophobic PVDF-co-HFP polymer, it neutralized the hydrophobic nature of the membrane and increased the hydrophilicity of the membrane. This shows that the carrier improved the membrane by reducing the hydrophobicity properties. Correspondingly, less hydrophobic properties of the membrane can facilitate the transfer of the MG cations across the membrane and in the meantime prevents the diffusion of aqueous solution into the receiving phase. The increment of the carrier in the membrane allows the surface of the membrane to have more interaction with the aqueous solution, thus increasing the transfer rate of the cations. The results are supported by the contradictory trend of water uptake of the membrane. M1 has the lowest water uptake of 2.02% followed by a gradual increment for M2 (13.43%), M3 (18.43%), M4 (20.99%), M5 (40.80%) and M6 (58.02%). The membrane with a higher hydrophobic surface will have lower wettability and lower tendencies to absorb water [66]. Likewise, the water uptake of the PIMs can also be related to the vehicular and Grotthuss mechanism [67]. During water absorption, the water molecules in the distilled water form hydrogen bonds with the active sites of the carriers, and hence, the higher composition of the carrier would provide more active sites for more water molecules retaining in the membrane. Moreover, immersing the membrane in water would increase the porosity of the membrane [68,69], and enhance the mobility of ions as well as the efficiency of the transportation of cations across the membrane [70]. In addition, from the SEM and AFM image, it can be observed that the carrier was embedded at the surface of the membrane which potentially reduces the interface energy [71]. Hence, the contact angle gradually reduced and increased the water uptake of the membrane.

### 3.6. Ion Exchange Capacity (IEC)

**Figure 12:**
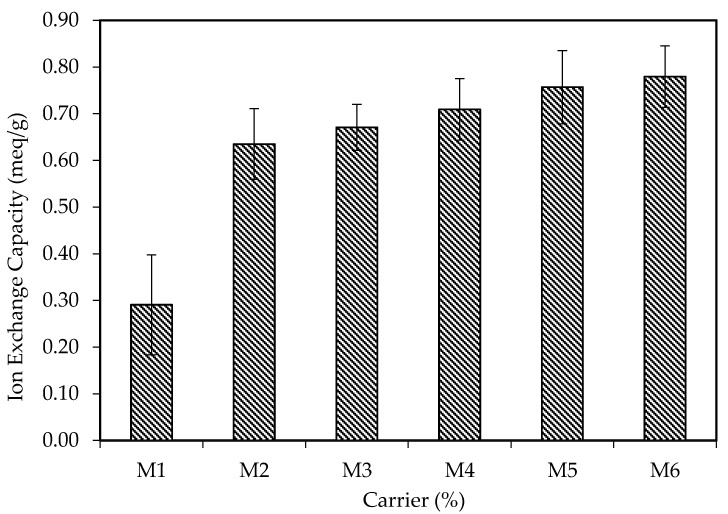
Ion exchange capacity (IEC) of M1, M2, M3, M4, M5 and M6.

Figure 12 shows the results of the IEC of M1, M2, M3, M4, M5 and M6. Based on Figure 12, it can be observed that M1 has the lowest IEC value of 0.291 meq/g; then the value surged to 0.635 meq/g for M2 and was followed by a steady increment in the IEC for the following membranes M3 (0.671 meq/g), M4 (0.709 meq/g), M5 (0.757 meq/g) and M6 (0.780 meq/g). The trend shows the relationship between the carrier content and IEC of the membrane, where the IEC increases in alliance with the carrier content of the membrane [72]. This is because the PIM with a higher percentage of carriers has more vacant ion-exchange sites available for the formation of carrier complexes between the carriers B_2_EHP and MG ions (Equation (9)). Thus, more MG ions can be extracted across the membrane into the receiving phase, and as a result, the IEC of the membrane was enhanced. However, the absence of carrier content significantly reduced the ion conductivity of the membrane. As proven by M1, the IEC value of M1 was the least among the membranes. Even though M1 does not contain any carrier, the membrane still tends to extract a small amount of MG. This is due to the presence of the –HFP amorphous phase of the semi-crystalline PVDF-co-HFP polymer which contributes a high dielectric constant (ɛ = 8.4) to the conductivity of the membrane [13,73]. Similar findings were also reported by other researchers on the enhancement of the ionic conductivity of the PVDF-co-HFP polymer with the presence of the –HFP group [74,75,76].

## 4. Kinetics Studies

### 4.1. Percentage of Carrier

Experimental data from the performance studies were simulated with pseudo-first-order (PFO) (Equation (6)) and pseudo-second-order (PSO) (Equation (7)). The results are presented in Table 7 and Table 8. The tables show the estimated parameter values of the PFO and PSO models for theoretical equilibrium concentration (*C_e_*), rate constant (k*_2_*), theoretical initial concentration (*C_0_*), correlation coefficient (*R^2^*) and variance obtained from the experimental data for each percentage of the carrier. Observably, the R^2^ value generated from the PFO is within the range of 0.9220 to 0.9365, whereas the range value for PSO is within the range of 0.9713 and 0.9997. By comparing both R^2^ values, it can be deduced that the experimental data have a better fit towards PSO in comparison with the PFO model. Therefore, the PFO model was used for subsequent discussion.

**Figure 13:**
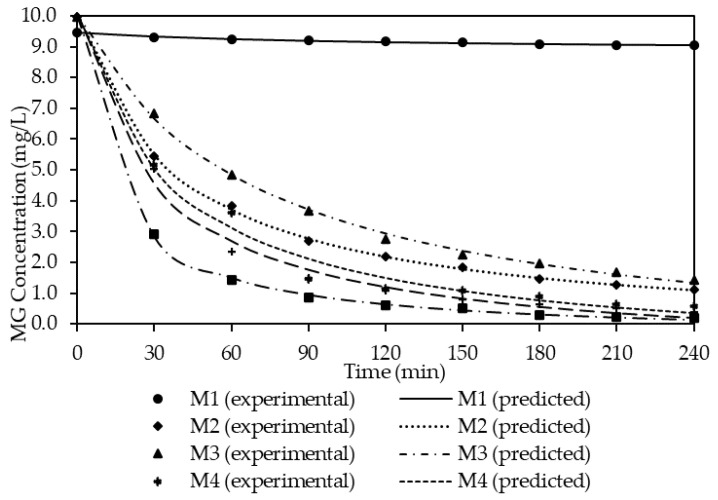
Comparison of the experimental data and simulations from PSO models with different percentages of the carrier, B_2_EHP.

Based on Table 8, it can be deduced that the rate of the extraction (*k_2_*) increases and the theoretical equilibrium concentration (*C_e_*) decreases as the percentages of the carrier increase. This can be supported by the results obtained for the ion-exchange capacity in Figure 12. The membrane with a higher percentage of carrier has higher ion capacity for the MG extraction; thus, more cation can be extracted before reaching the equilibrium state. The *R^2^* value is one of the most significant determinants and represents the good fit of experimental data and the model [77]. As evaluated, the R_2_ values for the PSO model are relatively high which fall within the range of 0.9713 to 0.9997. This shows that the experimental data for different percentages of the carrier are well fitted with the PSO model. This also indicates that the extraction mechanism of PIMs undergoes chemisorption mechanisms which involve oxidation and reduction processes [51,77,78,79]. As observed, M6 has the highest mass transfer coefficient, k_2_, and the lowest theoretical equilibrium constant, *C_e_*, of 0.0070 and 0.4235 mg/L, respectively. Hence, this proves that M6 is the most efficient membrane for the extraction of MG dye. In order to ease the analysis of the data, the experimental and theoretical data are presented in Figure 13.

### 4.2. Initial Dye Concentration

Experimental data from the performance studies were simulated with pseudo-first-order (PFO) (Equation (6)) and pseudo-second-order (PSO) (Equation (7)). The results are presented in Table 9 and Table 10. The tables show the estimated parameter values of the PFO and PSO models for theoretical equilibrium concentration (*C_e_*), rate constant (k*_2_*), theoretical initial concentration (*C_0_*), correlation coefficient (*R^2^*) and variance obtained from the experimental data at different dye concentrations. Observably, the R^2^ value generated from the PFO is within the range of 0.9213 and 0.9354, whereas the range value for PSO is within the range of 0.9985 and 0.9999. By comparing both R^2^ values, it can be deduced that the experimental data have a better fit towards PSO in comparison with the PFO model. Therefore, the PFO model was used for subsequent discussion.

In Table 10, the *k_2_* value decreases from 0.0340 to 0.0056 and the *C_e_* value increases from 0.1293 to 0.5361 with increasing initial dye concentration. This indicates that the increment in initial dye concentration affects the ability of the membrane to extract the MG ions across the membrane. At a higher concentration of initial dye concentration, the amount of active sites on the surface of the membrane limits the amount of MG ions extracted across the membrane. Eventually, the extraction process will reach a state where saturation occurs on the surface of the membrane and only a small amount of MG ion is extracted from the feeding phase. Hence, the *k_2_* value drops as the initial dye concentration inclines. Good fitting of the experimental data with the PSO model was determined with a relatively high *R_2_* value and low variance. The *R_2_* values deduced from the PSO model were 0.9995, 0.9998, 0.9989, 0.9985, 0.9996 and 0.9999 for the MG initial dye concentrations of 2 mg/L, 4 mg/L, 6 mg/L, 8 mg/L, 10 mg/L and 12 mg/L, respectively. Better fitting of the experimental data with the PSO kinetic model indicates that the rate-determining step in the MG extraction involves chemisorption, where the occurrence of the valence forces between the carrier and cation is possible.

**Figure 14:**
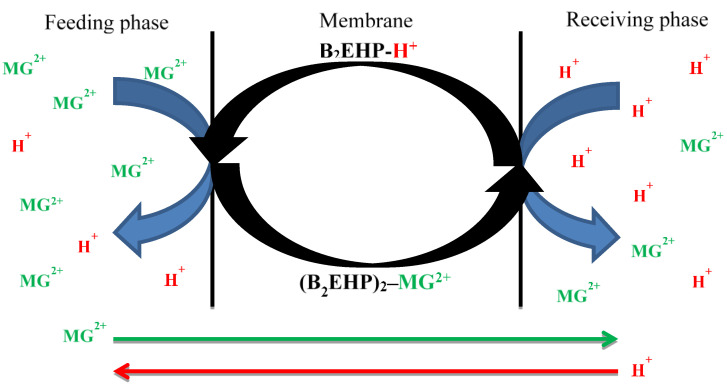
Mechanism of the MG extraction.

**Figure 15:**
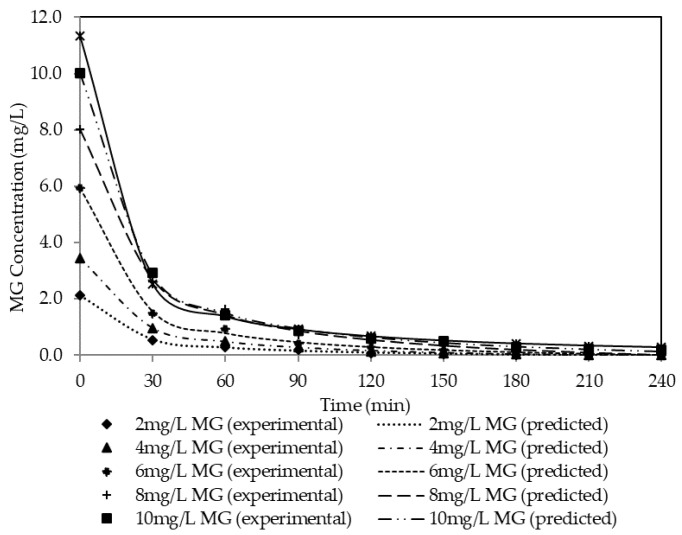
Comparison of the experimental data and simulations from PSO models at different dye concentrations.

Based on Motsoane [80], the mechanisms of the MG extraction involve three subsequent steps. First, the carriers undergo proton ionization, and protons are released into the feeding phase. Negatively charged compounds are formed. Then, the MG^2+^ ion is attracted to the carrier, forming a weak Van der Waals force of attraction and hydrogen bonds with the C-H and P-O-C groups found at the carrier. As a result, an ion-carrier complex is formed. Then, the MG^2+^ ion is transported across the membrane interface towards the receiving solution, and finally, the ion-carrier complex dissociated and released the MG^2+^ ion into the receiving solution. In exchange, the MG^2+^ ion is replaced by a proton (H^+^ ion) from the receiving solution, and the carrier is returned to the feeding solution where the extraction process is repeated. A high concentration of proton in the receiving phase tends to diffuse to the feeding phase with low proton concentration, down the concentration gradient. Therefore, this creates the driving force for the continuous extraction process, regardless of the concentration of the MG dye in the feeding phase. A similar occurrence was reported by Gherasim et al. [81] who deduced that the sorption mechanism of Pb(II) metal with PIMs containing the B_2_EHP carrier was based on the PSO kinetics model. Furthermore, Salima et al. [51] and Mahanty et al. [82] also concluded that PSO model can suitably describe the mechanism of extractions by PIMs. Figure 14 illustrates the mechanism of the MG extraction, while Figure 15 shows the plotting of the theoretical and experimental data *C_t_* vs. *t* of the extraction process at different MG concentrations.

## 5. Conclusions

The present study has successfully fabricated and characterized a new PVDF-co-HFP-based PIM with B_2_EHP as the carrier and DOP as the plasticizer. The membrane was applied in MG extraction which involves transporting the MG ions across the membrane from the feeding phase into the receiving phase.

Results show that the membrane with 21% of the B_2_EHP carrier achieved the best performance with 86.68% of MG extraction, 0.1188 cm/min of permeability, 1.1913 mg cm/min of flux and 78% IEC. This revealed that the performance of the membrane is dependent on the content of the carrier in the membrane. More than 97% of MG extraction was achieved within the selected range of initial dye concentration. Furthermore, the characterization of the membrane revealed that with the incorporation of the carrier, the surface of the membrane turned rougher, plus higher conductivity was achieved with a less hydrophobic surface. From the kinetic simulation of the experimental data, it was discovered that the trend of MG extraction was inclined towards PSO (R^2^ value >0.999) which subsequently described that the extraction took place via chemisorption mechanisms. This supports the idea where ions’ oxidation and reduction are involved in the process of MG extraction.

## Figures and Tables

**Table 1:** Examples of PIMs with different formulations and their applications.

Polymer Inclusion Membrane (PIM)				Extraction/Removal/Recovery	Extraction/Recovery/ Efficiency	Reference
Base Polymer	Plasticizer	Solvent	Carrier/ Extractant			
CTA	2-NPOE	DCM	Ester derivative of calix[4]arene (EDC)	MB	>90%	[14]
CTA	2-NPOE	DCM	Calixresorcin[4]arene derivative	Cr(IV)	98.4%	[15]
CTA	*o*-NPPE	DCM	Ethylenodiamino-bis-acetylacetone (EDAB-acac)	Zn(II)	90–98%	[16]
CTA	*o*-NPPE	DCM	Calix[4]pyrrole KP	Ag(I)	>92%	[17]
CTA	*o*-NPPE	DCM	Ethylenodiamino-bis-acetylacetone	Zn(II)	90%	[18]
				Cr(III)	65%	
				Ni(II)	6%	
CTA	NPOE	DCM	Aliquat 336	Picloram	97%	[19]
CTA	DOA	DCM	Dinonylnaphthalene sulfonic acid (DNNSA)	Co(II)	73.99%	[20]
CTA	-	CHCl_3_	Di-(2-hethylhexyl) phosphoric acid (D_2_EHPA)	Cu(II)	74%	[21]
CTA	*o*-NPPE	DCM	1-octyl-2,4-dimethylimidazole	Zn(II)	95.5%	[22]
CTA	NPOE	CHCl_3_	Trioctylmethylammonium Thiosalicylate (TOMATS)	Hg	84 ± 7%	[23]
CTA + PMMA PVC + PMMA	NPOE	CHCl_3_	-	Pb(II)	12.15% 25.31%	[24]
CTA/PBAT	-	CHCl_3_	Aliquat 336	Cr(VI)	>99%	[25]
PVC	B_2_EHP	THF	2,6-Diaminopyridine	Cu(II)	72.81%	[26]
				Zn(II)	93.65%	
PVC	DOP	THF	B_2_EHP	MG MB	97%	[27]
PVC	NPOE	THF	2-hydroxy-5- nonyl-benzaldoxime (M5640)	Cu(II)	100%	[28]
PVC	-	THF	1-octanol (OCT)	Phenol	82.8 wt%	[29]
PVDF	2-NPOE	DMF	Aliquat 336	Cr(IV)	96.9%	[30]
PVDF	NPOE	DMA	Aliquat 366	Au(I)	96.4%	[31]
PVDF-co-HFP	-	THF	Aliquat 336	Reactive Orange 16	99.62%	[32]
PVDF-co-HFP	2-NPOE	THF	Cyphos IL101	Cr(IV)	95.9%	[33]
PVDF-co-HFP	-	THF	Trihexyltetradecylphosphonium chloride, Cyphos^®^ IL 101	Cr(IV)	~95%	[34]

Abbreviations: 2-nitrophenyl n-octyl ether (2-NPOE); 2-nitrophenyl octyle-ether (NPOE); Bis-(2-ethylhexyl) phosphate (B2EHP); Cellulose triacetate (CTA); Chloroform (CHCl3); Chromium (Cr); Cobalt (Co); Copper (Cu); Dichloromethane (DCM); Dimethylformamide (DMA); Dioctyl adipate (DOA); Gold (Au); Lead (Pb); Malachite green (MG); Mercury (Hg); Methylene Blue (MB); N,N-dimethylformamide (DMF); o-nitrophenyl pentyl ether (o-NPPE); Poli ε-caprolactone (PCL); Poly(butylene adipate-co-terephthalate (PBAT); Poly(vinylidene fluoride-co-hexafluoropropylene) (PVDF-co-HFP); Polymethyl methacrylate (PMMA); Polyvinyl chloride (PVC); Polyvinylidene fluoride (PVDF); Silver (Ag); Tetrahydrofuran (THF); Thermoplastic polymers polyurethane (TPU); Thiocyanate (SCN-); Zinc (Zn).

**Table 2:** Formulation of PIMs in this study.

Membrane	PVDF-co-HFP (wt%)	B_2_EHP (wt%)	DOP (wt%)	THF (wt%)
M1	18	0	0	82
M2	18	6	1	75
M3	18	9	1	72
M4	18	15	1	66
M5	18	18	1	63
M6	18	21	1	60

**Table 3:** Influence of the percentage of the carrier on permeability (*P*) and flux (*J*) of the membrane.

Membrane	*P* (cm/min)	*J* (mg cm/min)
M1	0.0068	0.0643
M2	0.0255	0.2540
M3	0.0306	0.3046
M4	0.0407	0.4082
M5	0.0492	0.4936
M6	0.1188	1.1913

**Table 4:** Influence of the initial dye concentration on permeability (*P*) and flux (*J*) of the membrane.

Initial Dye Concentration (mg/L)	*P* (cm/min)	*J* (mg cm/min)
2	0.5772	1.2283
4	0.3157	1.0929
6	0.2139	1.2721
8	0.1715	1.3741
10	0.1358	1.3615
12	0.0951	1.0791

**Table 5:** The values of the wavenumbers (cm^−1^) and some significant peaks in the FTIR spectra of the pure B_2_EHP, pure DOP and membranes (M1 and M6).

Materials	Wavenumbers (cm^−1^)	Type of Molecular Vibrations
B_2_EHP	2958.08–2860.00	sp^3^ C- H
	1684.05	O-H stretching
	1380.10	sp^3^ C-H bending
	1008.08	P-O-C
	727.52	C-H_2_ rocking
DOP	2957.76–2859.54	sp^3^ C-H stretching
	1723.52	C=O
	1599.91–1460.44	C-C stretch in ring
	1266.60	C-O stretching
	1119.17	C-O stretching
	770.34–740.95	sp^2^ C-H bend in aromatic
M1 (18 wt% PVDF-co-HFP, 72 wt% THF)	3025.80–2923.07	C-H_2_ symmetric and anti-symmetric stretching vibration
	1454.88–1064.79	C-F stretching
	974.67	C-F stretching
	871.09	C-C stretching
	795.70	C-F_3_ stretching vibration
	760.85	C-H_2_ rocking vibration
	612.49	C-F_2_ bending and C-C-C vibration
M6 (PVDF-co-HFP 18 wt%: B_2_EHP 21 wt%:DOP 1 wt%:THF 60 wt%)	2958.92–2860.40	sp^3^ C-H
	1728.32	C=O
	1462.78	C-C stretching in ring
	1424.20–1382.67	sp^3^ C-H bending
	1208.87–1149.83	C-F stretching
	1019.71	P-O-C
	872.58	C-C stretching
	795.59	C-F_3_ stretching vibration
	761.71	C-H_2_ rocking vibration
	727.45	C-H_2_ rocking
	612.8	C-F_2_ bending and C-C-C vibration

**Table 6:** Contact angle, thickness and water uptake of PIMs.

Membrane	Contact Angle (°)	Water Uptake (%)
M1	153.48 ± 2.56	2.02 ± 0.2
M2	124.94 ± 1.57	13.43 ± 0.2
M3	110.18 ± 0.07	18.43 ± 0.4
M4	99.40 ± 2.23	20.99 ± 0.4
M5	95.86 ± 1.11	40.80 ± 0.3
M6	95.54 ± 3.03	58.02 ± 0.4

**Table 7:** Estimated parameter values of the PFO models by the numerical calculations for the extraction process with different percentages of the carrier (B_2_EHP).

Membrane	*C_e_*	*k _1_*	*C_0_*	*R^2^*	Variance
M1	9.0060	0.0092	9.4468	0.9220	0.0009
M2	1.3056	0.0212	9.8472	0.9249	0.0556
M3	1.2576	0.0219	9.9393	0.9296	0.0045
M4	0.5984	0.0222	9.9793	0.9328	0.0952
M5	0.5081	0.0256	10.0391	0.9287	0.0182
M6	0.4125	0.0419	9.9916	0.9365	0.0463

**Table 8:** Estimated parameter values of the PSO models by the numerical calculations for the extraction process with different percentages of the carrier (B_2_EHP).

Membrane	*C_e_*	*k_2_*	*C_0_*	*R^2^*	Variance
M1	8.8711	0.0004	9.4595	0.9713	0.0007
M2	1.3216	0.0015	9.9722	0.9996	0.0039
M3	1.2082	0.0018	10.0220	0.9997	0.0157
M4	1.1316	0.0024	10.0601	0.9987	0.1481
M5	1.0430	0.0029	10.0843	0.9997	0.0854
M6	0.4235	0.0070	10.0289	0.9996	0.0053

**Table 9:** Estimated parameter values of the PFO models by the numerical calculations for the extraction process at different dye concentrations.

Concentration	*C_e_*	*k _1_*	*C_0_*	*R_2_*	Variance
2	0.0465	0.0439	2.1177	0.9327	0.0046
4	0.0727	0.0433	3.4432	0.9245	0.0092
6	0.1797	0.0419	5.9157	0.9313	0.0418
8	0.2475	0.0404	7.9416	0.9213	0.0756
10	0.4125	0.0344	9.9916	0.9265	0.0463
12	0.5468	0.0331	11.3278	0.9354	0.0784

**Table 10:** Estimated parameter values of the PSO models by the numerical calculations for the extraction process at different dye concentrations.

Concentration	*C_e_*	*k_2_*	*C_0_*	*R_2_*	Variance
2	0.1293	0.0340	2.1276	0.9995	0.0003
4	0.2375	0.0186	3.4612	0.9998	0.0003
6	0.2972	0.0126	5.9448	0.9989	0.0051
8	0.3093	0.0101	8.0106	0.9985	0.0133
10	0.4235	0.0080	10.0289	0.9996	0.0053
12	0.5361	0.0056	11.3504	0.9999	0.0006

## Data Availability

The data presented in this study are openly available in the results and discussion section of this study.
